# Fabrication of a Dual-Drug-Loaded Smart Niosome-g-Chitosan Polymeric Platform for Lung Cancer Treatment

**DOI:** 10.3390/polym15020298

**Published:** 2023-01-06

**Authors:** Atefeh Zarepour, Abdurrahim Can Egil, Melike Cokol Cakmak, Monireh Esmaeili Rad, Yuksel Cetin, Seyma Aydinlik, Gozde Ozaydin Ince, Ali Zarrabi

**Affiliations:** 1 Biomedical Engineering Department, Faculty of Engineering & Natural Sciences, Istinye University, Istanbul 34396, Türkiye; 2 Faculty of Engineering and Natural Sciences, Materials Science and Nano-Engineering Program, Sabanci University, Istanbul 34956, Türkiye; 3 Nanotechnology Research and Application Center (SUNUM), Sabanci University, Tuzla 34956, Türkiye; 4 TUBITAK Marmara Research Center, Life Sciences Medical Biotechnology, Gebze 41470, Türkiye; 5 TUBITAK Marmara Research Center, Life Sciences, Industrial Biotechnology, Gebze 41470, Türkiye; 6 Center of Excellence for Functional Surfaces and Interfaces (EFSUN), Sabanci University, Istanbul 34956, Türkiye

**Keywords:** niosome, Rose Bengal, dual responsive nanocarrier, anticancer, antibacterial

## Abstract

Changes in weather conditions and lifestyle lead to an annual increase in the amount of lung cancer, and therefore it is one of the three most common types of cancer, making it important to find an appropriate treatment method. This research aims to introduce a new smart nano-drug delivery system with antibacterial and anticancer capabilities that could be applied for the treatment of lung cancer. It is composed of a niosomal carrier containing curcumin as an anticancer drug and is coated with a chitosan polymeric shell, alongside Rose Bengal (RB) as a photosensitizer with an antibacterial feature. The characterization results confirmed the successful fabrication of lipid-polymeric carriers with a size of nearly 80 nm and encapsulation efficiency of about 97% and 98% for curcumin and RB, respectively. It had the Korsmeyer–Peppas release pattern model with pH and temperature responsivity so that nearly 60% and 35% of RB and curcumin were released at 37 °C and pH 5.5. Moreover, it showed nearly 50% toxicity against lung cancer cells over 72 h and antibacterial activity against *Escherichia coli*. Accordingly, this nanoformulation could be considered a candidate for the treatment of lung cancer; however, in vivo studies are needed for better confirmation.

## 1. Introduction

According to global estimations, lung cancer is known as the third most common type of cancer and the first leading cause of cancer-related death with a five-year survival rate of about 18.6%. It is usually divided into two main types: non-small-cell lung carcinoma (NSCLC) with an abundance of about 80% and small-cell lung carcinoma (SCLC), which accounts for the remaining 20% of lung cancer. Daily enhancement of air pollution along with the changes in peoples’ lifestyles such as increasing smoking and changing in food habits have led to an elevated number of patients with lung cancer and risk of mortality. The main therapeutic method used for the treatment of diagnosed cases of lung cancer includes a combination of chemotherapy, radiotherapy, or immunotherapy with surgery. Among the different treatment methods, chemotherapy is preferred due to its effects on the prevention of cancer recurrence and metastasis; however, most chemotherapeutic agents are hydrophobic compounds that could not reach to their targeted site [1,2,3]. Accordingly, it is important to introduce novel methods that could enhance the efficacy of treatment.

**Figure 1:**
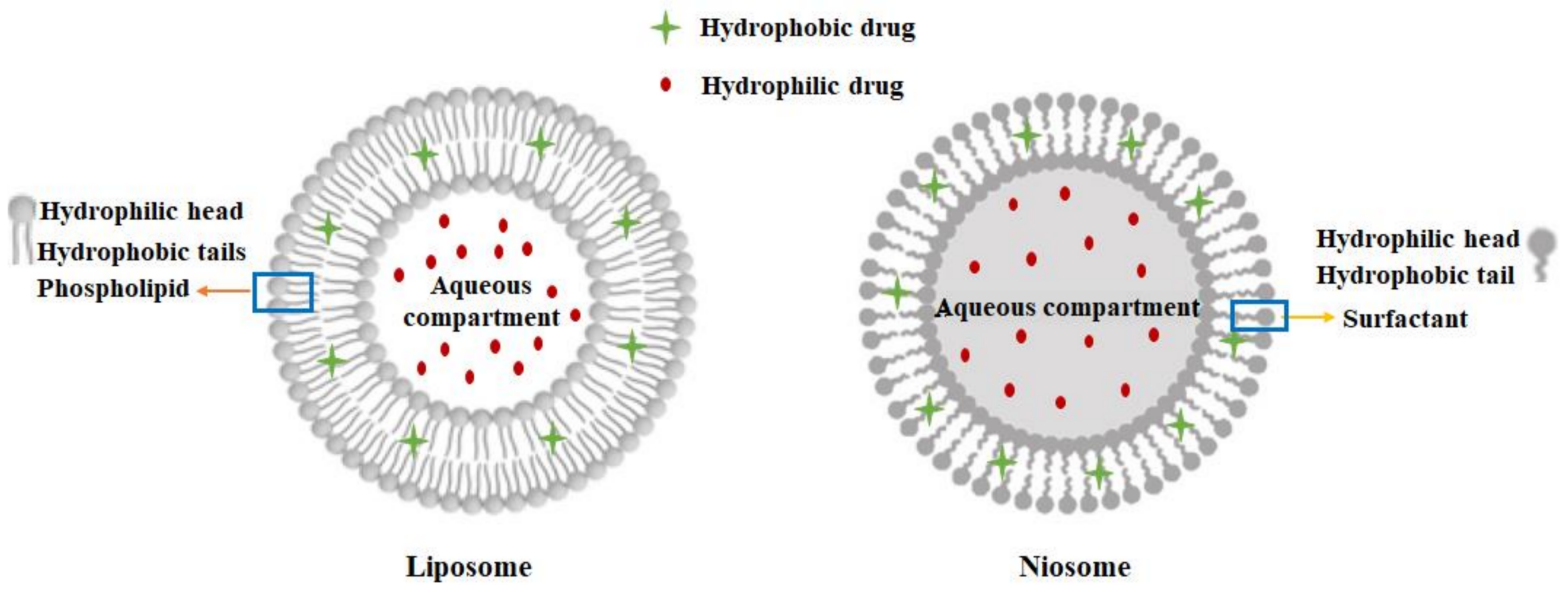
The similarity and differences of liposomal and niosomal structures. These are spherical nanostructures with bilayer membranes that have the capability of delivering both hydrophobic and hydrophilic therapeutic agents. However, niosomes are composed of surfactants, which are cheaper than phospholipids.

Recent studies focus on the design of different types of targeted delivery nanoformulations to overcome the low water solubility of the therapeutic agents, enhancing their bioavailability in their targeted site, decreasing the administrated dose of the drug, and reducing their side effects on normal cells [4,5]. Niosome nanocarriers are one type of these nanocarriers with a spherical shape that are fabricated from a bilayer nonionic surfactant. Like liposomes, niosomes are composed of hydrophilic heads and lipophilic tails, having the capability of carrying hydrophobic and hydrophilic therapeutic agents simultaneously (Figure 1). These are biodegradable and biocompatible carriers with lower cost of production when compared to liposomes; however, they have a drawback, namely, drug leakage, that could limit their effectiveness in their targeted site [6,7]. To overcome this limitation, surface functionalization of the niosomes with polymeric ligands such as polyethylene glycol (PEG) and chitosan have been suggested, leading to the fabrication of stealth niosome [8,9]. Indeed, the application of polymeric materials such as chitosan, PEG, poly(N-vinyl-2-pyrrolidone), poly(vinyl alcohol), polycaprolactone, and poly lactic glycolic acid, in the structure of nanoformulation could enhance their loading efficiency, bioavailability, and therapeutic performance for different applications [10,11,12].

Despite the effectiveness of these polymeric functionalizations on prevention of drug leakage from the niosome during its journey to the targeted cells, they could decrease the performance of drug release in the targeted site. This limitation can be overcome by using responsive agents that respond to decreased pH or increased temperature of the cancer tissues. This leads to the fabrication of smart stealth niosomes that have the capability of releasing most of the drug molecules in their targeted site rather than normal tissues [13].

Cancerous tissues are also prone to bacterial infections, complicating the treatment process. For example, one of the interesting features of lung cancer is the presence of bacterial infections in the cancerous tissues. Indeed, it is revealed that cancer cells change the microbiota of lung to increase the inflammation and promotion of cancer. Different types of bacteria were determined that have a relationship with the creation and promotion of lung cancer; among one group of them is Gram-negative bacteria such as *Escherichia coli* (*E. coli*) [14,15]. Thus, the addition of antibacterial compounds in the drug-loaded carriers could further enhance the efficacy and prevent the inflammation reaction.

Rose Bengal (RB) or 4,5,6,7-tetrachloro-2′,4′,5′,7′-tetraiodofluorescein is a dye with a photodynamic antibacterial property that was firstly used as a wool and food dye in the 19th century in Japan. It is a type of photosensitizer that can produce reactive oxygen species (ROS) under irradiation by light at a wavelength of 550 nm via the energy transfer pathway. It also could enhance the antibacterial activity of other compounds such as β-caryophyllene through a photooxidation reaction. RB could also show its antibacterial activity via acting as a sonosensitizer compound, in dark conditions, via absorbing the electromagnetic energy and transferring it into the heat [16,17,18,19,20].

This study, therefore, is aimed at synthesizing and evaluating the antibacterial and anticancer ability of a new type of smart nanocarrier. To reach this aim, curcumin, as a type of anticancer drug model, was chosen and loaded inside the niosome. Then, these niosomal carriers were functionalized by a chitosan polymer that not only has the capability of creating the responsiveness feature for a new nanoformulation but also could enhance their bioavailability in the lung cancer tissue via promoting their attachments to the mucus due to its muco-adhesive property [21,22] and improving cell attachment due to its positive surface charge. Rose Bengal, as a photosensitizer agent with antibacterial activity, was also loaded inside the carrier. Then, different types of physicochemical analysis including DLS, FE-SEM, TEM, FTIR, and Zeta potential were implemented to evaluate the fabrication of the nanoformulation. In order to confirm the responsiveness of the carrier against pH and temperature, the drug release patterns were examined at two different pH values (5.5 and 7.4) and two temperatures (25 °C and 37 °C). Furthermore, the kinetic of the release was evaluated, employing five different kinetic models. In the final step, the antibacterial and anticancer properties of the formulation were assessed against two different types of bacteria (*E. coli* and *Staphylococcus aureus* (*S. aureus*)) and A549 lung cancer cells.

## 2. Materials and Methods

### 2.1. Materials

Sorbitan monostearate (Span 60) and Tween 20 were purchased from Alfa Aesar (Massachusetts, United States). Cholesterol was purchased from PanReac AppliChem (Darmstadt, Germany). Chloroform (99.8%), methanol (99.9%), chitosan (75–85% deacetylated, medium molecular weight), N-vinyl-caprolactam, N, N′-methylenebisacrylamide (MBA), ammonium persulfate (APS), Rose Bengal, acetic acid, fetal bovine serum (FBS), penicillin–streptomycin antibiotic, trypsin–EDTA, and curcumin (65%) were from Merck (Darmstadt, Germany). Dulbecco’s modified Eagle’s medium (DMEM) culture media and phosphate-buffered saline (PBS, pH 7.4) were purchased from PAN-Biotech (Aidenbach, Germany). Luria–Bertani (LB) broth media and Luria–Bertani (LB) were obtained from Sigma-Aldrich (Missouri, United States). A549 lung carcinoma epithelial cell line and bacterial strains (Escherichia coli (E. coli, ATCC strain: 25,922) and Staphylococcus aureus (S. aureus, ATCC strain: 29,213)) were supplied from ATCC (Virginia, USA). Milli-Q water was used for all the experiments.

### 2.2. Preparation of Curcumin Loaded Niosomes

The thin-film method was used for the preparation of curcumin-loaded niosome [23]. Briefly, Span 60, cholesterol, and curcumin (with a 2:1:1 molar ratio) were dissolved in a mixed solution of chloroform and methanol (50 mL, with 1:3 volume ratio) and then evaporated using a rotary vacuum evaporator (Heidolph, Schwabach, Germany) at 60 °C and 150 RPM. Then, phosphate-buffered saline (PBS) was added to the fabricated thin film layer, and sample was sonicated at 60 °C for 1 h to fabricate nanoniosome-loaded curcumin. The stabilization process was conducted via overnight storage at 4 °C. The final products were washed and centrifuged at 4 °C and 12,000 RPM to remove the unloaded curcumin. Bare niosomes were prepared with the same process in the absence of curcumin.

### 2.3. Preparation of Chitosan-Coated Niosome Nanoparticles

#### 2.3.1. Grafted Polymer Fabrication

**Scheme 1:**
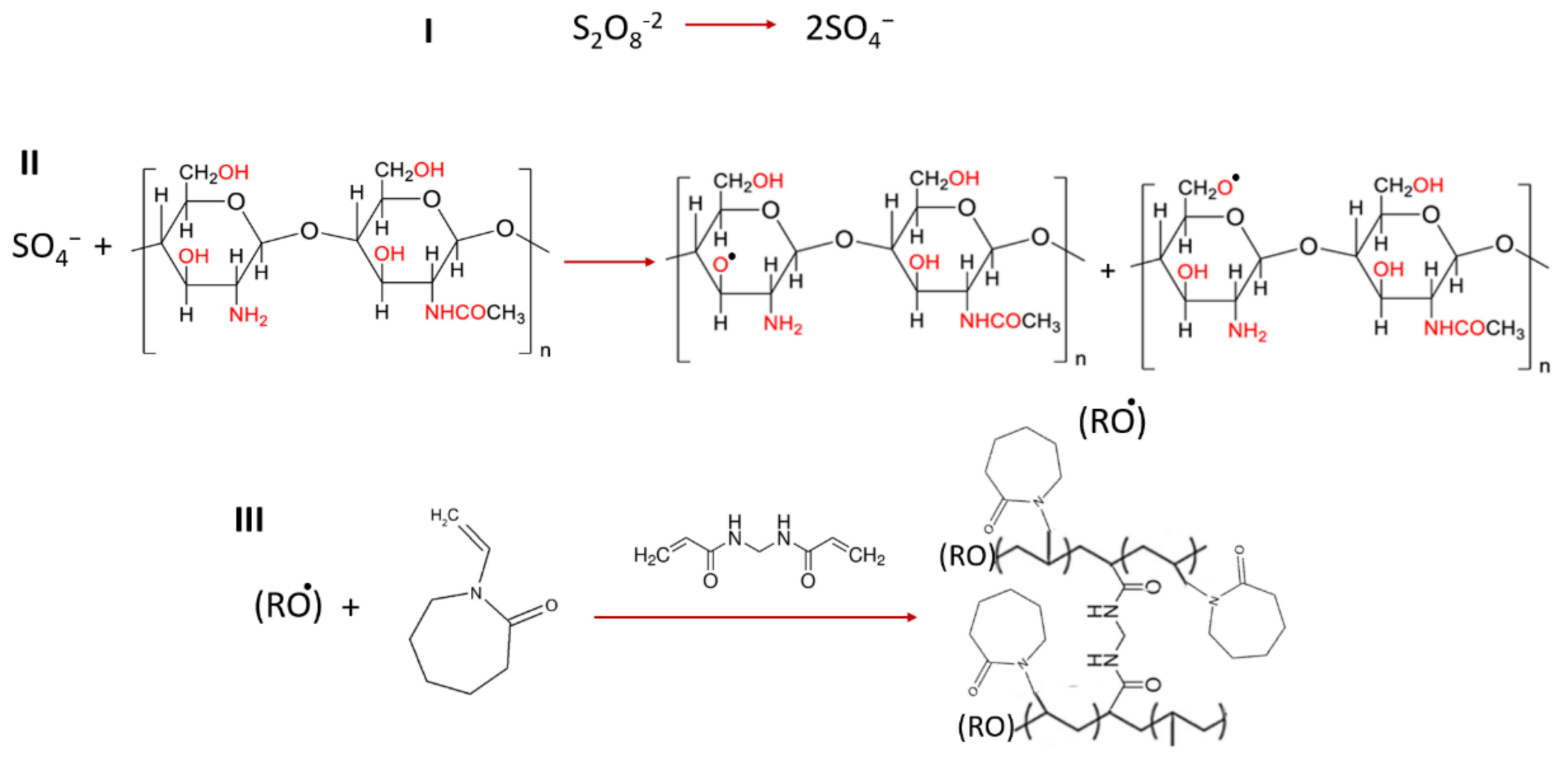
Chemical reactions related to the preparation of grafted polymer.

The grafting of N-vinyl-caprolactam (NVCL) on the chitosan was performed on the basis of the procedure of Duan et al., with small modifications [24]. Briefly, a proper amount of chitosan was dissolved in 1% acetic acid solution, and the pH value was adjusted to pH = 5.0. Following this, the solution was heated to 70 °C under nitrogen, and APS (1.5 mL, 1.0 × 10^−2^ mol/L) was added to it. Then, sodium bicarbonate buffer (NaHCO₃) was added to the reaction system to maintain a constant pH value of the reaction mixture, preventing hydrolysis of n-vinyl-caprolactam at acidic conditions. After 15 min, NVCL and MBA (as crosslinker) were added, and the mixture was stirred for 3 h under nitrogen. The resulting polymer solution was dialyzed (molecular weight cut off (MWCO) 12 kDa) against Ultrapure Milli Q double-distilled water for 7 days and then freeze dried (ScanVac CoolSafe Pro 100-9 (LaboGene, Lillerød, Denmark)) (Scheme 1).

#### 2.3.2. Fabrication of CSgPVCL-Coated Niosomes

The coating process was performed using a microfluidic syringe pump (KD Scientific Legato 100). An equal volume of 1% (*w*/*v*) aqueous polymer solution containing a predetermined amount of Rose Bengal was added dropwise at a 25 µL/s rate to the curcumin-loaded niosome solution. The polymer–niosome mixtures were stirred for 1 h at room temperature and then centrifuged at 17,000 RPM for 30 min to remove the excess polymer and unencapsulated Rose Bengal molecules. The same process was used to prepare a drug-free sample.

### 2.4. Physicochemical Characterization

#### 2.4.1. Size and Zeta Potential

The hydrodynamic size, surface charge, and polydispersity index (PDI) of the nanoparticles were determined using a ZetaSizer Nano ZS (Malvern Instruments, Worcestershire, UK) instrument, equipped with a 4.0 mV He-Ne laser (633 nm) at 25 °C. The hydrodynamic size of each sample was measured at 25 °C, and their related standard deviation was determined after a threefold repetition.

#### 2.4.2. Electron Microscopy

Morphology and size of the nanoparticles were assessed by a field emission scanning electron microscope (FE-SEM) (Zeiss Leo Supra 35 VP SEM-FEG, Jena, Germany) at a 3 kV operating voltage. Nanoparticles (10 µL) were dropped on a piece of the silicon wafer, dried, and coated with Au-Pd using a sputter coater (Cressington 108, Cressington Scientific Instruments Ltd, Watford, UK) at 40 mA for 120 s. A secondary electron (SE) detector was used to obtain the FE-SEM images. Moreover, 3 µL of stock solution was dropped on a transmission electron microscopy (TEM) grid and analyzed at 200 kV using the TEM microscope (JEM-ARM200, JEOL, MA, USA).

#### 2.4.3. Fourier Transform Infrared (FTIR) Spectroscopy

FTIR analysis (Thermo Scientific Nicolet iS10, MA, USA) was used in the range of 4000–650 cm^−1^ to confirm the correct preparation of polymeric shell with a deuterated triglycine sulfate detector.

### 2.5. Determination of Encapsulation Efficiency and Loading Capacity of the Nanoparticles

Encapsulation efficiency (EE%) and loading capacity (LC%) were evaluated using a UV-visible spectrophotometer. In the case of curcumin, after preparing niosomes, they were centrifuged to precipitate the drug-loaded niosomes, and then the supernatant was separated and the precipitated niosomes were washed and centrifuged two more times to remove the physically adsorbed drug molecules. Then, the amounts of unloaded drug (which were presented inside the supernatant) were determined via UV-visible spectroscopy at a wavelength of 425 nm, and the percentages of EE and LC were determined using Equations (1) and (2) [25]. The same process was applied after encapsulating curcumin-loaded niosomes and RB inside the polymeric shell, and the amounts of unloaded RB were determined after measuring the adsorption of the supernatant at 560 nm.

(1) $$EE\%=\frac{Total\;amount\;of\;drug-Amount\;of\;unloaded\;drug}{Total\;amount\;of\;drug}\times 100$$

(2) $$LC\%=\frac{Total\;amount\;of\;drug-Amount\;of\;unloaded\;drug}{Total\;amount\;of\;nanocarrier}\times 100$$

### 2.6. Drug Release Test and Release Kinetics Analysis

The drug-released pattern of the polymer-coated niosomes were determined at two different pH values (7.4 as body normal pH and 5.5 as endosomal pH) and two different temperatures (25 °C and 37 °C, as storage temperature and body temperature, respectively). To this end, drug-loaded nanoparticles were put in dialysis capsules with a cellulose membrane (MWCO 12 kDa). The capsules were placed in beakers containing 50 mL of PBS (contained 5% Tween 20, with two different pH values) and incubated in a shaker incubator. After certain interval times (1, 3, 6, 12, 24, 48, 72, 96, and 120 h), 3 mL of each solution was replaced by fresh PBS (3 mL), and the absorbances of the isolated samples were determined via UV–VIS analysis at 425 nm and 560 nm to determine the release amount of curcumin and RB, respectively [26]. Then, the resulting release profiles were analyzed using several kinetic models, and the results were compared through their *R*^2^ values.

### 2.7. Cytotoxicity Assay

The anticancer property of the fabricated nanocomplex was evaluated via MTT assay [27]. In detail, A549 cells (human lung carcinoma epithelial cell line) were cultured at a cell density of 1 × 10^4^ cells/well in DMEM as cultured media completed with 10% FBS and 1% antibiotic (penicillin–streptomycin). Cells were incubated for 24 h at 37 °C with 5% CO_2_. Then, the cultured media were replaced with fresh media containing different concentrations (25, 50, 100, and 200 µg.mL^−1^) of nanoparticles with/without drug molecules (complete complex and bare complex, respectively). Cells treated with fresh media were used as a negative control, and free curcumin was used as a positive control. After 24, 48, and 72 h incubation at 37 °C, the media of each well were removed, cells were washed with PBS (twice), and 100 µL of fresh media containing 10 µL of MTT solution (with concentration of 5 mg/mL in PBS) was added to each well; following this, the plates were incubated for 4 h in the dark at 37 °C. During this process, the MTT salts were converted to formazan via an enzymatic reaction conducted by the mitochondria of the viable cells. After that, the media of the wells were replaced by 100 µL DMSO (to solubilize the precipitated formazan), and plates were incubated for another 1 h. Finally, the absorbance of each well was measured by a microplate reader (BioTek, Vermont, USA) at 570 nm, and the reference wavelength 650 nm and the percentage of viable cells were determined by comparing the results of each concentration with that of the control.

### 2.8. Antibacterial Test

Antibacterial properties of the synthesized nanoparticles with/without drug were assessed against two pathogenic bacteria: *Escherichia coli* (*E. coli*) and *Staphylococcus aureus* (*S. aureus*). For this, the bacterial strains were grown in LB broth medium for 18 h on a 200 RPM shaker at 37 °C. Nanoparticles with the concentration of about 2.4 mg.mL^−1^ in PBS (pH = 7.4) were prepared and sterilized using UV irradiation. Then, a volume of 5 µL of bacterial cell cultures (OD_600_ = 0.1) were added to 500 µL of nanoparticle solution and incubated for 24 h at 37 °C. After that, 100 µL of each solution was spread onto the surface of Luria–Bertani (LB) agar plates using sterile glass beads, and the plates were incubated for another 24 h at 37 °C. The bacterial colonies grown on the plates were observed and compared. PBS media and free Rose Bengal were used as negative and positive controls, respectively [28].

### 2.9. Statistical Analysis

SPSS software (version 21, parametric analysis of variance, ANOVA (Tukey), IBM, New York, USA) was applied for quantitative evaluation of the results. The significancy was determined via *p* ≤ 0.05.

## 3. Results

### 3.1. Characterization of Nanoparticles

The average size, surface charge, and dispersity index of bare niosomes, curcumin-loaded niosomes, and chitosan-coated niosomes containing both curcumin and Rose Bengal were determined using Zeta sizer (Table 1). As be seen in the Table 1, the size of niosomes was increased after encapsulating curcumin inside these nanoparticles. Indeed, the hydrophobic nature of curcumin leads to its location between the two layers of the niosome membrane, its hydrophobic part, leading to an increase in the size of the niosomes. The interesting point is despite this increase in size, the PDI of the nanoparticles did not change significantly, which could confirm the structural stability of niosomes and their uniform size. After coating curcumin-loaded niosomes with the polymeric layer containing RB, the size of nanoparticles was increased, which could confirm the presence of a new polymeric shell. Moreover, the PDI of the particles was increased a little, which could represent distribution of the nanoparticles at nearly the same size.

In the case of surface potential, encapsulating the drug led to a slight increase in the charge of nanoparticles. Surface charge of niosomes confirmed their liquid stability due to their high negative charge. Indeed, it is revealed in the literature that nanoparticles with charge more than ±30 mV are stable in liquid solution [29]. Interestingly, coating with chitosan polymer led to a significant change in the surface charge of particles from −46.1 to +30.7 mV. This positive charge could have resulted from the presence of the amine groups in the structure of chitosan and NVCL compounds. This high positive charge of the polymer-coated niosomes also confirmed their liquid stability.

**Figure 2:**
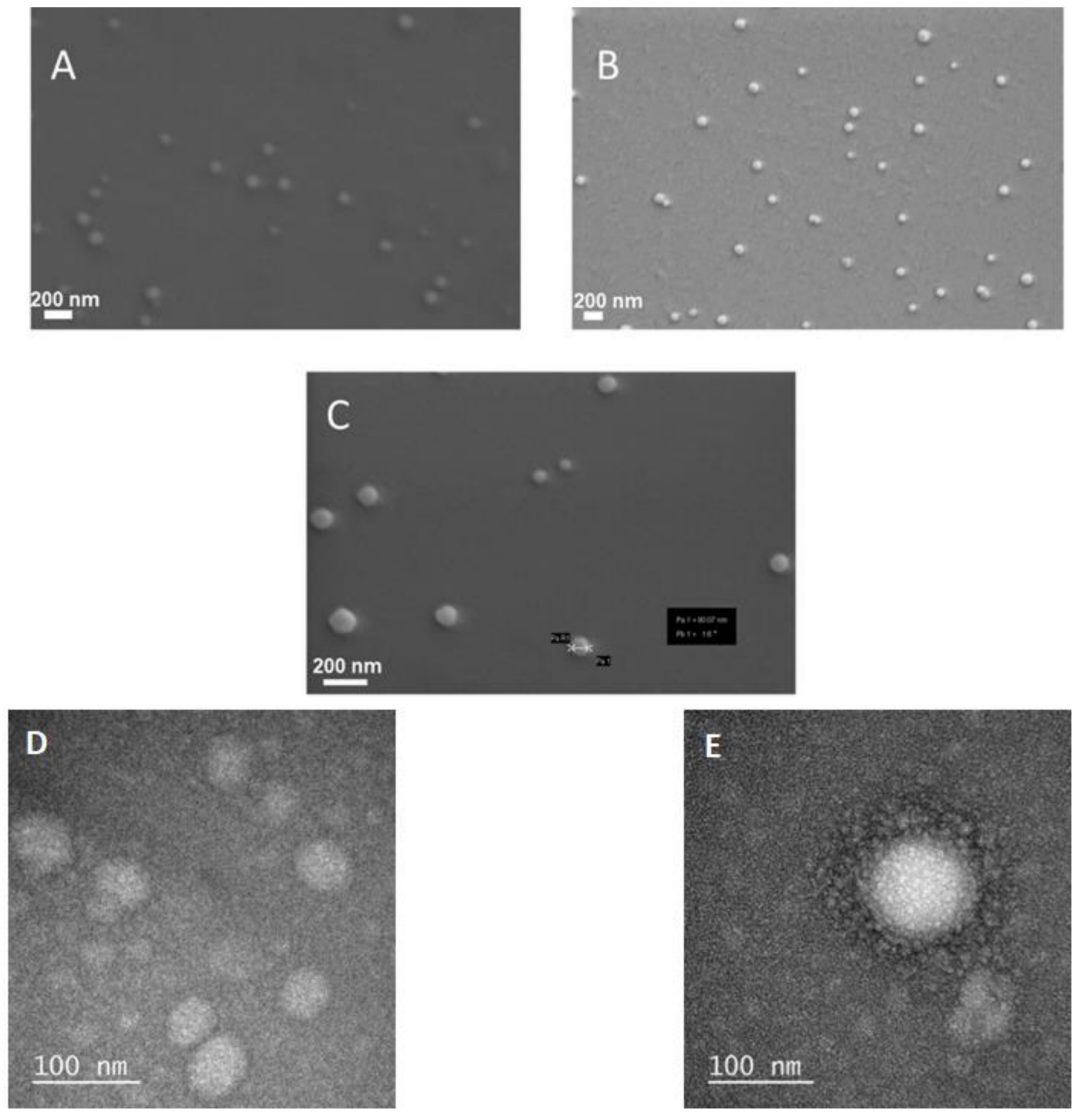
Electron microscopy images of different nanoparticles. FESEM image of (**A**) bare niosome, (**B**) curcumin-loaded niosomes, (**C**) chitosan-coated niosome with curcumin and Rose Bengal. TEM images of (**D**) bare niosome and (**E**) chitosan coated niosome with curcumin and Rose Bengal.

Electron microscopies (TEM and FESEM) were used to determine the size and morphology of nanoparticles. Figure 1 shows the SEM and TEM results of bare niosomes, curcumin-loaded niosomes, and polymer-coated niosomes with both curcumin and Rose Bengal. The results of the SEM images (Figure 2A–C) confirmed the spherical shape of nanoparticles, even after coating with chitosan polymers with a size of about 80 nm, being similar to the results of DLS. Moreover, the size obtained from the TEM images confirmed the results of DLS tests (Figure 2D,E).

**Figure 3:**
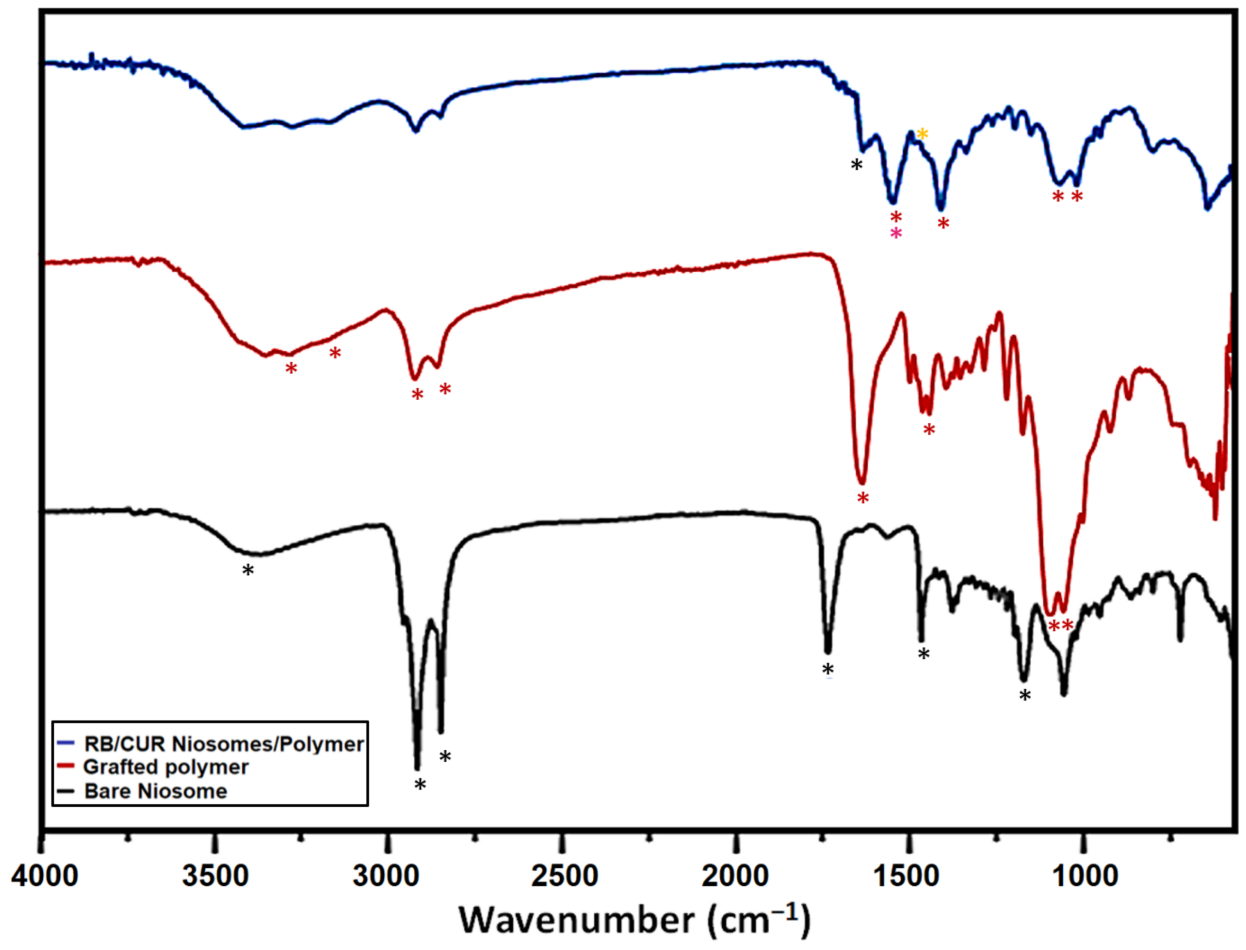
FTIR spectrums of bare niosome, grafted polymer, and complete complex (RB/CUR niosome/polymer). Asterisks with different colors are used to distinguish bands on different compounds. Black (*), Red (*), Orange (*), and Pink (*) are attributed to the bands of niosome, grafted polymer, curcumin, and RB, respectively.

FTIR analysis was used to determine the chemical composition of the synthesized particles. Figure 3 shows the FTIR results of bare niosome, grafted polymer, and complete complex (polymer coated niosome loaded with both drugs). The bands between 2800 and 2900 cm^−1^ and the band around 3400 cm^−1^ in the spectra were related to the stretching vibrations of alkyl (C-H and C-H_2_) and hydroxyl groups, respectively. These bands existed in the carrier, grafted polymer, and coated carrier. In the spectrum of bare niosome, the C=C stretch from the cholesterol structure and C=O and C-O stretching of ester groups of Span60 were observed at 1564, 1734, and 1243 cm^−1^, respectively. In the spectrum of the grafted polymer, the band at 1690 cm^−1^ was related to the C=O stretching of the amid bond of chitosan. Moreover, two bands at around 1050 and 1100 cm^−1^ in the spectrum of grafted polymer were attributed to C-O stretching of the primary and secondary alcohols of chitosan. Moreover, bands between 3100 and 3300 cm^−1^ were attributed to the primary amin presented in the structure of chitosan and secondary amin presented in the structure of the crosslinker, MBA. The band related to the C=O group of NVCL appeared at around 1478 cm^−1^. The last spectrum shows the curcumin-loaded niosome coated by the grafted polymer incorporating RB. In the spectrum of the complete complex, there was a shift in the stretching bands related to C=O groups of niosome (from 1734 to 1700 cm^−1^), grafted polymer (from 1690 to 1600 cm^−1^), primary (from 1100 to 1050 cm^−1^) and secondary alcohols (from 1050 to 990 cm^−1^) of chitosan, and the C=O group of NVCL (from 1478 to 1400 cm^−1^). Moreover, some of the bands related to curcumin and RB were also visible in the spectrum of the complete complex. For example, the olefinic CH group of curcumin confirmed by the weak band at around 1490 cm^−1^ (since curcumin is encapsulated inside niosome, it was predicted that we would see a very weak shifted band) and the C=O vibration of the RB carboxylic acid group at around 1600 cm^−1^, all of which confirmed the presence of drugs, niosomes, and polymeric compounds in the structure of the final product [23,24,30,31,32].

### 3.2. In Vitro Evaluation of Drug Loading and Released Pattern

The amounts of loading capacity and encapsulation efficiency of nanoparticle were calculated for both drugs, and the results are mentioned in Table 2. According to the results of the drug-loading test, nanoparticles showed a high amount of encapsulation efficiency for both drugs.

**Figure 4:**
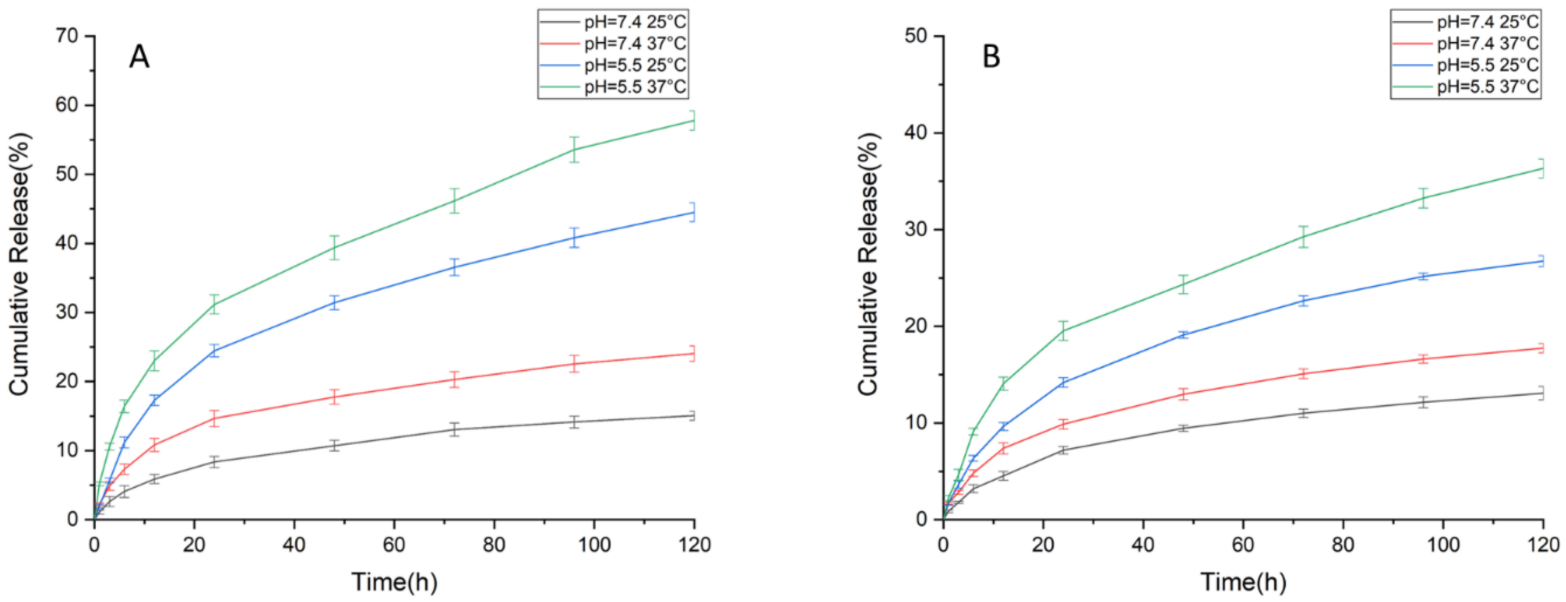
Drug release profile of (**A**) Rose Bengal and (**B**) CUR at two pH values and two temperatures.

The nanoparticles were exposed to two different pH value (pH = 7.4 and pH = 5.5) and temperature (25 °C and 37 °C) environments. The release profiles were depicted on the basis of cumulative release (%) and time (Figure 4). At all pH values and temperature levels, sustained-release profiles were observed, and thanks to the presence of temperature and a pH-responsive polymeric shell, faster drug release was achieved at an acidic level (pH = 5.5) and at 37 °C. This phenomenon can be attributed to the protonated amine groups of chitosan and NVCL, leading to swelling of the polymer at slightly acidic conditions and providing more space for drug diffusion. Moreover, at elevated temperature, the conformational change in the structure of polymer results in the shrinking of the polymer and promotes faster drug release [33,34].

According to the results, 57 % of Rose Bengal was released at pH = 5.5 and 37 °C, whereas this amount was 24% at pH = 7.4 and the same temperature. On the other hand, curcumin showed a drug release of about 36% and 17% at pH 5.5 and 7.4, respectively. The pH responsiveness of the resulting nanoparticles displayed faster drug release at acidic pH levels. Moreover, at 25 °C, the released amount of Rose Bengal was about 44% at pH = 5.5 and only 15 % at pH = 7.4, showing the highly tunable feature of this nanoformulation. Curcumin showed 26% and only 13% in the same respective situations. In addition to pH sensitivity, the temperature-responsive release behavior of this nanoparticle is attributed to the presence of a temperature-sensitive polymer, PNVCL, in the structure. Qian et al. prepared chitosan-g-PNIPAM nanocarriers and observed faster paclitaxel release at a higher temperature and acidic conditions (37 °C and pH= 5.5) than at room temperature and physiological pH conditions (25 °C and pH= 7.4) [35]. Moreover, Chen et al. also proposed to have a pH-sensitive drug release of hydrophilic doxorubicin (DOX) and hydrophobic hydroxycamptothecin (HCPT) from a single nanocarrier including one drug at the core and the other drug interacting with the polymeric shell. They observed faster release at acidic pH and sustained release at physiological pH conditions for both drug molecules [36].

### 3.3. Evaluation of Drug Release Kinetic

Following the curve fitting of all release data (Table 3), the Korsmeyer–Peppas model had the highest R^2^ value (>0.97) among the other kinetic analysis models. The *n* values for each release profile were above 0.43, which indicates non-Fickian diffusion. In the Korsmeyer–Peppas model for spherical geometry, the drug release was coherent with Fickian diffusion in the case of the *n* value below 0.43, and this means that the diffusion rate was greater than the polymeric chain relaxation process. In the other extreme condition where *n* was greater than 0.85, drug release was governed by swelling or relaxation of polymeric chains, and this phenomenon was also correlated with zero-order kinetics. In addition to these two extreme conditions, the non-Fickian or in other words the anomalous model was observed, wherein the *n* value was between 0.43 and 0.85. In this case, the drug release mechanism is governed by both diffusion and swelling [37]. In addition to the Korsmeyer–Peppas model, our drug release profiles had good correlation factors with the Higuchi model, although our system did not satisfy the model requirement of negligible swelling/dissolution of the matrix or higher initial drug concentration in the matrix than the solubility of the drug molecules [38].

On the other hand, first-order release kinetics states that the release rate only depends on the concentration; the Hixson–Crowell model assumes that the drug release is related to dissolution velocity and is not related to diffusion [39]. In consideration of our R^2^ values for these two models, it can be clearly seen that our release profiles were not coherent with them as well.

### 3.4. Anticancer Activity Test

**Figure 5:**
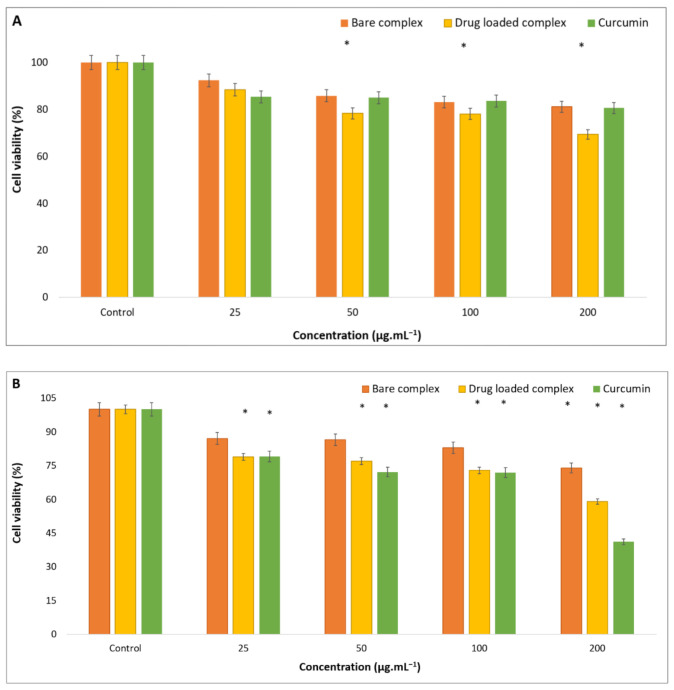

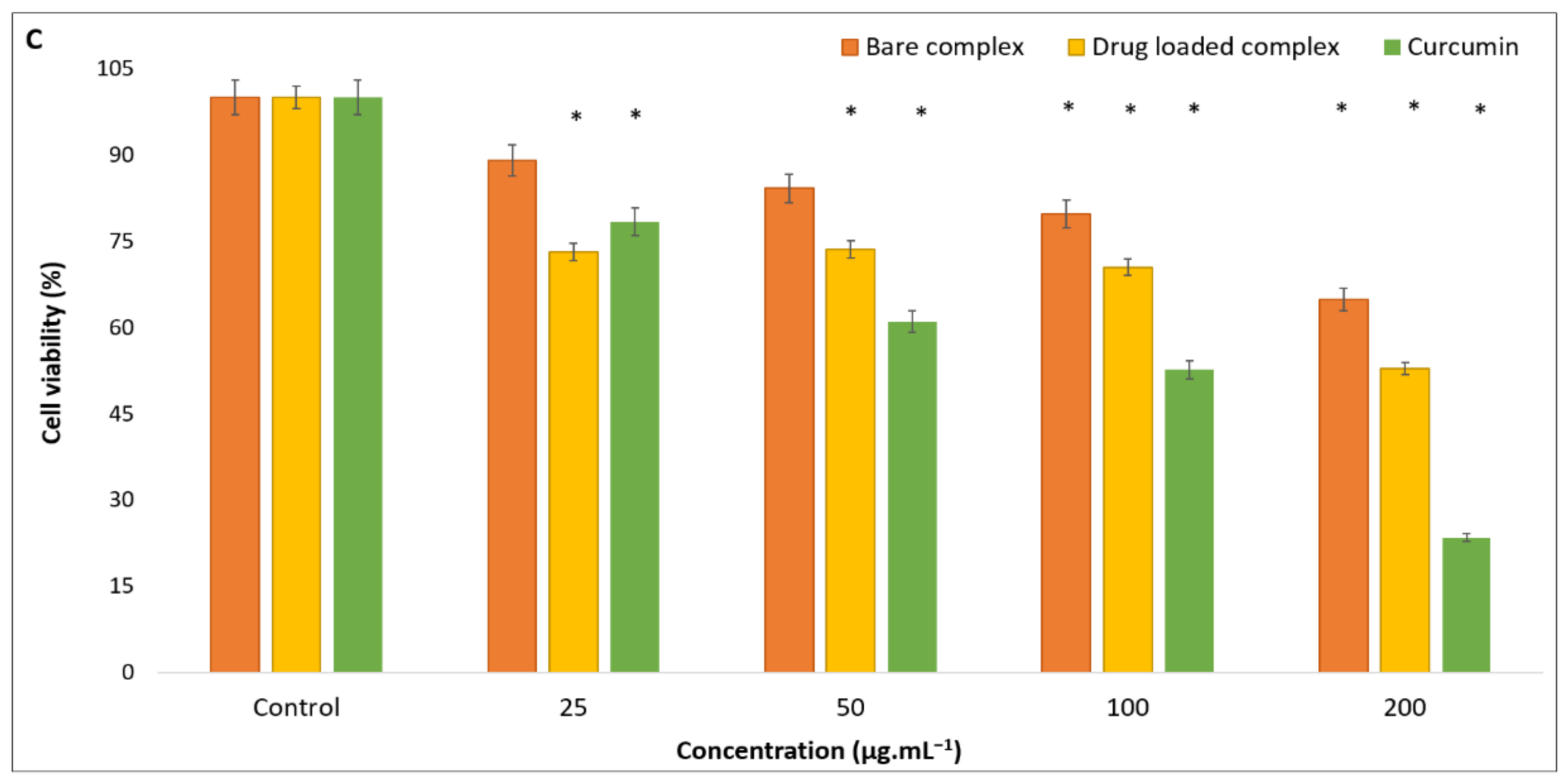
The results of the MTT assay test of different formulations after 24 h (**A**), 48 h (**B**), and 72 h (**C**) exposure with the A549 cancer cell line (* indicates *p* ≤ 0.05).

The anticancer properties of the fabricated nanoformulation were determined via MTT cytotoxicity assay (Figure 5). After evaluating the results with SPSS software, it was concluded that during the first 24 h of exposing cells with different compounds, by increasing the concentration from 25 to 200 µg.mL^−1^, no significant toxicity was observed for bare nanoformulation that could confirm the biocompatibility of bare formulation. In the case of the drug-loaded carrier, cytotoxicity was increased by increasing the concentration so that near 31% toxicity was achieved at the concentration of 200 µg.mL^−1^ against cancer cells, while the free drug had no significant effect. We suggest that utilizing the nanocarrier led to improving the water solubility and bioavailability of the anticancer curcumin that resulted in cellular internalization of the drug and thus anticancer effects exhibition. Conversely, in the case of the drug alone, its low solubility could prevent its cellular internalization, and thus no significant cytotoxicity was achieved. This could confirm the effectiveness of a drug-loaded nanoformulation for cancer treatment applications. By increasing the exposing time and due to the decreasing the tampon ability of the culture media, the pH of media decreased, that could enhance the drug release from the nanoparticles and enhance the cytotoxicity effects. Accordingly, after 48 and 72 h, nearly 22% and 27% cytotoxicity effects were seen for the lowest concentration of drug-loaded nanoformulation, respectively. In the highest concentration, this cytotoxicity effect reached about 42 and 48% (for 48 and 72 h, respectively). Moreover, we could see toxicity for some of the concentrations of the bare complex after 48 h, which could have been due to the interactions occurring between positively charged chitosan and negatively charged components of the cancer cells that could have destructive effects on cells. Moreover, as could be seen in the figure, below 200 µg/mL, there was no significant differences between the cytotoxicity of free and loaded drug after 48 h; however, the differences were increased after 72 h so that there was a significant difference in the lower concentrations as well.

### 3.5. Antibacterial Test

**Figure 6:**
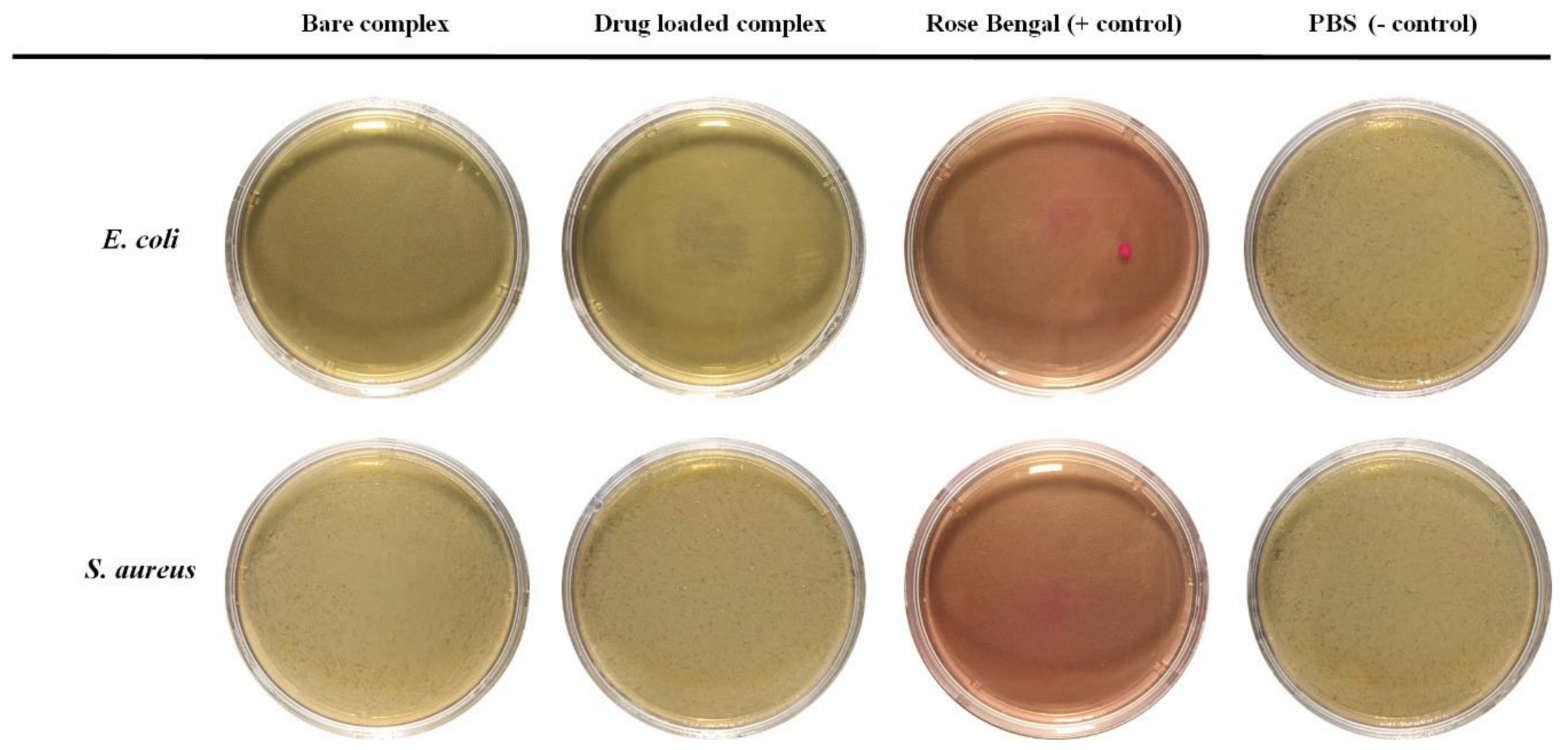
The results of the antibacterial test of the bare complex, drug-loaded complex, and Rose Bengal against two types of bacteria: the Gram-negative bacteria *E. coli* and the Gram-positive bacteria *S. aureus*.

The antibacterial activity of polymer (chitosan)-coated niosome with/without curcumin and RB (drug-loaded complex and bare complex) on *E. coli* (Gram-negative) and *S. aureus* (Gram-positive) bacteria were evaluated using the colony method (Figure 6). As it is clear in the figure, the bare complex was found to be effective on *E. coli* even in the absence of any therapeutic compounds that could be related to the presence of chitosan. RB alone also showed antibacterial activity against both types of bacterial strains, while the drug-loaded complex had complete antibacterial activity and bacterial inhibition only against *E. coli*. This could be a good result in the case of lung cancer application since Gram-negative bacteria could affect cancer promotion.

## 4. Discussion

Lung cancer is the first leading cause of cancer-related death in the world, with a poor five-year survival rate resulting from the inefficiency of the current therapeutic method. The most prevalent method used for treatment of this cancer is chemotherapy, which shows low performance due to the low bioavailability of chemotherapeutic agents. On this basis, utilizing a delivery vehicle that could protect and enhance the bioavailability of these therapeutic agents in their targeted site could improve their performance [40]. Accordingly, in this study, we introduced a new type of drug delivery vehicle fabricated from niosome nanoparticles functionalized by a chitosan polymer. Moreover, curcumin was chosen as a type of anticancer agent. Briefly, curcumin-loaded niosome was fabricated at first via the thin-film hydration method, and then chitosan grafted with N-vinyl-caprolactam was coated on the surface of these niosomes in order to improve their stability. Furthermore, Rose Bengal, as a photosensitizer with antibacterial activity, was loaded on the surface of the niosomes during their coating with chitosan.

Chitosan was chosen to enhance the bioavailability of the nanoformulation in the lung and prevent their elimination via mucociliary clearance due to its ability to attach on the surface of the mucus layer and penetrate into deeper parts. This could provide the capability of utilizing the inhalation route for the administration of anticancer drugs (nasal drug delivery), which could also be more efficient since it can reach its targeted site in less time. It is a biocompatible polymer that could be metabolized by the lysosomal enzymes, and due to its positive charge, it could enhance the cellular uptake of the nanoformulation by the cancer cells via interacting with the negative charge of the cell membrane [41,42].

Initiation and promotion of lung cancer are accompanied by changes in the microbiota of the lung. Most changes that occur in the number of bacterial strains are related to *Granulicatella, Streptococcus*, and *Veillonella*; however, other types of bacteria such as some of the Gram-negative strains are mentioned as well [43]. On this basis, we added Rose Bengal into the structure of our nanoformulation, with it showing antibacterial activity via producing reactive oxygen species (ROS) when exposed to light irradiation. It also was able to inhibit the activity of the NAD^+^ and NADP^+^ dehydrogenase, as well as DNA/RNA polymerase [44]. The antibacterial activity of free RB against both Gram-positive and Gram-negative bacteria was confirmed in previous research [45]. The results of antibacterial tests of this study showed that both bare composite and dual-drug-loaded carrier have antibacterial activity that result from chitosan and chitosan + RB, respectively. This antibacterial activity could be attributed to the interaction of nanoparticles with the bacteria surface that is more common in Gram-negative bacteria compared to Gram-positive ones. Indeed, it is revealed in previous research that the positively charged compounds have antimicrobial effects only against the adsorbed Gram-negative bacteria [46]. In order to confirm that the antibacterial activity of a dual-drug-loaded carrier is related to both chitosan and RB, the antibacterial activity of nanoformulation without RB was also tested. Interestingly, in this test, the nanoformulation containing curcumin did not show an antibacterial effect (Figure S1). Although in some research, curcumin itself has antibacterial activity [47,48], in this study, no antibacterial activity obtained from this compound, which could be due to its low concentration.

In the case of anticancer activity, this nanocarrier showed good anticancer effects during 24–72 h, meaning that 200 µg.mL^−1^ of it was able to show nearly 31, 42, and 48% cytotoxicity against lung cancer cells (after 24, 48, and 72 h, respectively). As mentioned previously, this nanoformulation is a type of pH-responsive agent that could show higher amounts of drug release in lower pH; however, it is not possible to completely provide such a pH value in in vitro tests due to the presence of the tampon in the media. Indeed, during the culturing time, cells uptake nutrition from the media and release their metabolites into it, leading to the media changing color to yellow, showing the acidic pH of the media. Moreover, on the basis of the results of the release test, such a good cytotoxicity effect was related to about 30% of loaded curcumin (after 72 h), which could be a good result in comparison to the free drug. Accordingly, the cytotoxicity results could confirm the effectiveness of this nanoformulation on decreasing the dose of the drug for the required treatment, which is important for preventing the formation and promotion of the drug resistance feature in cancer cells. In comparison to other works that used curcumin against the lung cancer cells, the toxicity results of this study showed similar or better performance [49,50,51].

## 5. Conclusions

In summary, in this study, a dual-drug-loaded smart carrier was fabricated via encapsulating curcumin-loaded niosome and Rose Bengal inside a chitosan-g-PVCL shell in a spherical shape with a mean diameter of 80 nm. The average PDI of 0.364 and average zeta potential of +30.7 mV confirmed the stability and size uniformity of the nanoparticles inside the solution. The fabricated nanocarriers showed high entrapment efficiency for both types of drugs and a pH and temperature release pattern. They also exhibited good antibacterial and anticancer effects against Gram-negative bacteria and lung cancer cells, respectively. All the above-mentioned features confirm the capability of this new nanoformulation as a potential candidate for the treatment of lung cancer.

## Figures and Tables

**Table 1:** Average size (based on the number average), polydispersity index, and zeta potential of niosomes (with and without curcumin) and polymer-coated niosomes with two different drugs.

Formulation	Size (nm)	PDI	Zeta Potential (mV)
Bare niosome	41 ± 7	0.316	−43.9
Curcumin-loaded niosome	56 ± 15	0.327	−46.1
Complete complex	80 ± 13	0.364	+30.7

**Table 2:** Encapsulation efficiency and loading capacity for curcumin and Rose Bengal co-encapsulated inside the nanoparticles.

Type of Drug	EE%	LC%
Curcumin	97.19%	7.67%
Rose Bengal	98.21%	8.61%

**Table 3:** Kinetic model analysis of the drug release profiles.

Formulation/Model	Korsmayer–Peppas		Higuchi	Zero Order	First Order	Hixson–Crowell
(RB/CUR)	(*R*^2^)	(*n*)	(*R*^2^)	(*R*^2^)	(*R*^2^)	(*R*^2^)
**RB1**	0.9943	0.46	0.9615	0.8835	0.6018	0.7204
**RB2**	0.9806	0.48	0.9705	0.9148	0.6888	0.7816
**RB3**	0.9954	0.46	0.9806	0.8955	0.6581	0.7569
**RB4**	0.9730	0.47	0.9234	0.8685	0.6406	0.7306
**CUR1**	0.9962	0.49	0.9810	0.9032	0.6720	0.7683
**CUR2**	0.9962	0.47	0.9808	0.9053	0.6499	0.7537
**CUR3**	0.9954	0.46	0.9797	0.8970	0.6694	0.7635
**CUR4**	0.9858	0.5	0.9727	0.8909	0.6825	0.7650

**RB1:** Rose Bengal release profile at pH = 5.5 and 25 °C, **RB2:** Rose Bengal release profile at pH = 5.5 and 37 °C, **RB3:** Rose Bengal release profile at pH = 7.4 and 25 °C, **RB4:** Rose Bengal release profile at pH = 7.4 and 37 °C, **CUR1:** curcumin release profile at pH = 5.5 and 25 °C, **CUR2:** curcumin release profile at pH = 5.5 and 37 °C, **CUR3:** curcumin release profile at pH = 7.4 and 25 °C, **CUR4:** curcumin release profile at pH = 7.4 and 37 °C.

## Data Availability

Not applicable.

## Supplementary Materials

The following supporting information can be downloaded at: https://www.mdpi.com/article/10.3390/polym15020298/s1, Figure S1: Effects of Curcumin loaded niosome-chitosan nanoparticles and free Curcumin on *E.coli* and *S.aureus*.

## References

[1] Lee R., Choi Y.J., Jeong M.S., Park Y.I., Motoyama K., Kim M.W., Kwon S.-H., Choi J.H. (2020). Hyaluronic acid-decorated glycol chitosan nanoparticles for pH-sensitive controlled release of doxorubicin and celecoxib in nonsmall cell lung cancer. Bioconjugate Chem..

[2] Saravanakumar K., Sathiyaseelan A., Mariadoss A.V.A., Jeevithan E., Hu X., Shin S., Wang M.-H. (2020). Dual stimuli-responsive release of aptamer AS1411 decorated erlotinib loaded chitosan nanoparticles for non-small-cell lung carcinoma therapy. Carbohydr. Polym..

[3] Zhang L., Wang J., Zhang Y., Ke L., Lin X., Li Z., Chen H., Gao Y. (2019). Indocyanine green-encapsulated erlotinib modified chitosan nanoparticles for targeted chemo-photodynamic therapy of lung cancer cells. Dye. Pigment..

[4] Almutairi F.M., Abd-Rabou A.A., Mohamed M.S. (2019). Raloxifene-encapsulated hyaluronic acid-decorated chitosan nanoparticles selectively induce apoptosis in lung cancer cells. Bioorganic Med. Chem..

[5] Zhu X., Yu Z., Feng L., Deng L., Fang Z., Liu Z., Li Y., Wu X., Qin L., Guo R. (2021). Chitosan-based nanoparticle co-delivery of docetaxel and curcumin ameliorates anti-tumor chemoimmunotherapy in lung cancer. Carbohydr. Polym..

[6] Aparajay P., Dev A. (2022). Functionalized niosomes as a smart delivery device in cancer and fungal infection. Eur. J. Pharm. Sci..

[7] Takzare A., Ghafoor D.D., Siddiqi A.F., Ravali S., Shalbaf M., Bakhtiar M. (2019). Trachyspermum copticum essential oil incorporated niosome for cancer treatment. J. Drug Deliv. Sci. Technol..

[8] Hemati M., Haghiralsadat F., Yazdian F., Jafari F., Moradi A., Malekpour-Dehkordi Z. (2019). Development and characterization of a novel cationic PEGylated niosome-encapsulated forms of doxorubicin, quercetin and siRNA for the treatment of cancer by using combination therapy. Artif. Cells Nanomed. Biotechnol..

[9] Witika B.A., Bassey K.E., Demana P.H., Siwe-Noundou X., Poka M.S. (2022). Current Advances in Specialised Niosomal Drug Delivery: Manufacture, Characterization and Drug Delivery Applications. Int. J. Mol. Sci..

[10] Kurniawan A., Gunawan F., Nugraha A.T., Ismadji S., Wang M.-J. (2017). Biocompatibility and drug release behavior of curcumin conjugated gold nanoparticles from aminosilane-functionalized electrospun poly (N-vinyl-2-pyrrolidone) fibers. Int. J. Pharm..

[11] Kurniawan A., Muneekaew S., Hung C.-W., Chou S.-H., Wang M.-J. (2019). Modulated transdermal delivery of nonsteroidal anti-inflammatory drug by macroporous poly (vinyl alcohol)-graphene oxide nanocomposite films. Int. J. Pharm..

[12] Mangindaan D., Chen C.-T., Wang M.-J. (2012). Integrating sol–gel with cold plasmas modified porous polycaprolactone membranes for the drug-release of silver-sulfadiazine and ketoprofen. Appl. Surf. Sci..

[13] Karimifard S., Rezaei N., Jamshidifar E., Moradi Falah Langeroodi S., Abdihaji M., Mansouri A., Hosseini M., Ahmadkhani N., Rahmati Z., Heydari M. (2022). pH-responsive chitosan-adorned niosome nanocarriers for co-delivery of drugs for breast cancer therapy. ACS Appl. Nano Mater..

[14] Jin C., Lagoudas G.K., Zhao C., Bullman S., Bhutkar A., Hu B., Ameh S., Sandel D., Liang X.S., Mazzilli S. (2019). Commensal microbiota promote lung cancer development via γδ T cells. Cell.

[15] Kovaleva O.V., Romashin D., Zborovskaya I.B., Davydov M.M., Shogenov M.S., Gratchev A. (2019). Human lung microbiome on the way to cancer. J. Immunol. Res..

[16] Kim Y., Park S., Lee E., Cerbo R., Lee S., Ryu C., Kim G., Kim J., Ha Y. (2008). Antibacterial compounds from rose Bengal-sensitized photooxidation of β-caryophyllene. J. Food Sci..

[17] Kurosu M., Mitachi K., Yang J., Pershing E.V., Horowitz B.D., Wachter E.A., Lacey III J.W., Ji Y., Rodrigues D.J. (2022). Antibacterial Activity of Pharmaceutical-Grade Rose Bengal: An Application of a Synthetic Dye in Antibacterial Therapies. Molecules.

[18] Li Y., Wang J., Yang Y., Shi J., Zhang H., Yao X., Chen W., Zhang X. (2021). A rose bengal/graphene oxide/PVA hybrid hydrogel with enhanced mechanical properties and light-triggered antibacterial activity for wound treatment. Mater. Sci. Eng. C.

[19] Nakonechny F., Barel M., David A., Koretz S., Litvak B., Ragozin E., Etinger A., Livne O., Pinhasi Y., Gellerman G. (2019). Dark antibacterial activity of rose Bengal. Int. J. Mol. Sci..

[20] Shrestha A., Hamblin M.R., Kishen A. (2014). Photoactivated rose bengal functionalized chitosan nanoparticles produce antibacterial/biofilm activity and stabilize dentin-collagen. Nanomed. Nanotechnol. Biol. Med..

[21] Babu A., Amreddy N., Muralidharan R., Pathuri G., Gali H., Chen A., Zhao Y.D., Munshi A., Ramesh R. (2017). Chemodrug delivery using integrin-targeted PLGA-Chitosan nanoparticle for lung cancer therapy. Sci. Rep..

[22] Li X., Wang J., Cui R., Xu D., Zhu L., Li Z., Chen H., Gao Y., Jia L. (2020). Hypoxia/pH dual-responsive nitroimidazole-modified chitosan/rose bengal derivative nanoparticles for enhanced photodynamic anticancer therapy. Dye. Pigment..

[23] Rad M.E., Egil A.C., Ince G.O., Yuce M., Zarrabi A.J.C., Physicochemical S.A., Aspects E. (2022). Optimization of curcumin loaded niosomes for drug delivery applications. Colloids Surf. A Physicochem. Eng. Asp..

[24] Duan C., Zhang D., Wang F., Zheng D., Jia L., Feng F., Liu Y., Wang Y., Tian K., Wang F. (2011). Chitosan-g-poly (N-isopropylacrylamide) based nanogels for tumor extracellular targeting. Int. J. Pharm..

[25] Paknia F., Mohabatkar H., Ahmadi-Zeidabadi M., Zarrabi A. (2022). The convergence of in silico approach and nanomedicine for efficient cancer treatment; in vitro investigations on curcumin loaded multifunctional graphene oxide nanocomposite structure. J. Drug Deliv. Sci. Technol..

[26] Damiri F., Bachra Y., Bounacir C., Laaraibi A., Berrada M. (2020). Synthesis and characterization of lyophilized chitosan-based hydrogels cross-linked with benzaldehyde for controlled drug release. J. Chem..

[27] Zarepour A., Zarrabi A., Larsen K.L. (2019). Fabricating β-cyclodextrin based pH-responsive nanotheranostics as a programmable polymeric nanocapsule for simultaneous diagnosis and therapy. Int. J. Nanomed..

[28] Cui H., Siva S., Lin L. (2019). Ultrasound processed cuminaldehyde/2-hydroxypropyl-β-cyclodextrin inclusion complex: Preparation, characterization and antibacterial activity. Ultrason. Sonochem..

[29] Raval N., Maheshwari R., Kalyane D., Youngren-Ortiz S.R., Chougule M.B., Tekade R.K., Rakesh K.T. (2019). Importance of physicochemical characterization of nanoparticles in pharmaceutical product development. Basic Fundamentals of Drug Delivery.

[30] Egil A.C., Carmignani A., Battaglini M., Sengul B.S., Acar E., Ciofani G., Ince G.O. (2022). Dual stimuli-responsive nanocarriers via a facile batch emulsion method for controlled release of Rose Bengal. J. Drug Deliv. Sci. Technol..

[31] Islam S., Arnold L., Padhye R. (2015). Comparison and characterisation of regenerated chitosan from 1-butyl-3-methylimidazolium chloride and chitosan from crab shells. BioMed Res. Int..

[32] Kozanoǧlu S., Özdemir T., Usanmaz A. (2011). Polymerization of N-vinylcaprolactam and characterization of poly (N-vinylcaprolactam). J. Macromol. Sci. Part A.

[33] Das S.S., Bharadwaj P., Bilal M., Barani M., Rahdar A., Taboada P., Bungau S., Kyzas G.Z. (2020). Stimuli-responsive polymeric nanocarriers for drug delivery, imaging, and theragnosis. Polymers.

[34] Xie Y., Tuguntaev R.G., Mao C., Chen H., Tao Y., Wang S., Yang B., Guo W. (2020). Stimuli-responsive polymeric nanomaterials for rheumatoid arthritis therapy. Biophys. Rep..

[35] Qian Q., Niu S., Williams G.R., Wu J., Zhang X., Zhu L.-M. (2019). Peptide functionalized dual-responsive chitosan nanoparticles for controlled drug delivery to breast cancer cells. Colloids Surf. A Physicochem. Eng. Asp..

[36] Chen X., Song L., Li X., Zhang L., Li L., Zhang X., Wang C. (2020). Co-delivery of hydrophilic/hydrophobic drugs by multifunctional yolk-shell nanoparticles for hepatocellular carcinoma theranostics. Chem. Eng. J..

[37] Bruschi M.L. (2015). Strategies to Modify the Drug Release from Pharmaceutical Systems.

[38] Paul D. (2011). Elaborations on the Higuchi model for drug delivery. Int. J. Pharm..

[39] Paarakh M.P., Jose P.A., Setty C., Christoper G. (2018). Release kinetics–concepts and applications. Int. J. Pharm. Res. Technol..

[40] Guo X., Zhuang Q., Ji T., Zhang Y., Li C., Wang Y., Li H., Jia H., Liu Y., Du L. (2018). Multi-functionalized chitosan nanoparticles for enhanced chemotherapy in lung cancer. Carbohydr. Polym..

[41] Al-Kinani M.A., Haider A.J., Al-Musawi S. (2021). Design, construction and characterization of intelligence polymer coated core–shell nanocarrier for curcumin drug encapsulation and delivery in lung cancer therapy purposes. J. Inorg. Organomet. Polym. Mater..

[42] Shim S., Yoo H.S. (2020). The application of mucoadhesive chitosan nanoparticles in nasal drug delivery. Mar. Drugs.

[43] Mur L.A., Huws S.A., Cameron S.J., Lewis P.D., Lewis K.E. (2018). Lung cancer: A new frontier for microbiome research and clinical translation. Ecancermedicalscience.

[44] Demartis S., Obinu A., Gavini E., Giunchedi P., Rassu G. (2021). Nanotechnology-based rose Bengal: A broad-spectrum biomedical tool. Dye. Pigment..

[45] Ferreira T.A.C., Warth J.F.G., Dos Santos L.L., Moore B.A., Montiani-Ferreira F. (2020). Antimicrobial activity of topical dyes used in clinical veterinary ophthalmology. Vet. Ophthalmol..

[46] Gottenbos B., Grijpma D.W., van der Mei H.C., Feijen J., Busscher H.J. (2001). Antimicrobial effects of positively charged surfaces on adhering Gram-positive and Gram-negative bacteria. J. Antimicrob. Chemother..

[47] Gao M., Long X., Du J., Teng M., Zhang W., Wang Y., Wang X., Wang Z., Zhang P., Li J. (2020). Enhanced curcumin solubility and antibacterial activity by encapsulation in PLGA oily core nanocapsules. Food Funct..

[48] Mahmud M.M., Zaman S., Perveen A., Jahan R.A., Islam M.F., Arafat M.T. (2020). Controlled release of curcumin from electrospun fiber mats with antibacterial activity. J. Drug Deliv. Sci. Technol..

[49] Dong Y., Yang Y., Wei Y., Gao Y., Jiang W., Wang G., Wang D. (2020). Facile synthetic nano-curcumin encapsulated Bio-fabricated nanoparticles induces ROS-mediated apoptosis and migration blocking of human lung cancer cells. Process Biochem..

[50] Hoshikawa A., Nagira M., Tane M., Fukushige K., Tagami T., Ozeki T. (2018). Preparation of curcumin-containing α-, β-, and γ-cyclodextrin/polyethyleneglycol-conjugated gold multifunctional nanoparticles and their in vitro cytotoxic effects on A549 cells. Biol. Pharm. Bull..

[51] Praveena J., Hakkimane S.S., Guru B.R. (2022). Synergistic effect of Paclitaxel and Curcumin in nano-formulations on U87 and A549 cancer cell lines. J. Appl. Pharm. Sci..

