# Supplementary material for: Fabrication of a Dual-Drug-Loaded Smart Niosome-g-Chitosan Polymeric Platform for Lung Cancer Treatment
Source: Polymers (Basel). 2023 Jan 6;15(2):298. doi: 10.3390/polym15020298 (PMC9860619; doi:10.3390/polym15020298)
Supplement: Supplementary file 1 [file polymers-15-00298-s001.zip › polymers-2085495-supplementary.pdf]

polymers

Supporting information

Fabrication of a Dual-Drug-Loaded Smart Niosome-g-Chitosan Polymeric Platform for Lung Cancer Treatment

Atefeh Zarepour [1], Abdurrahim Can Egil [2], Melike Cokol Cakmak [3], Monireh Esmaeili Rad [2,3], Yuksel Cetin [4], Seyma Aydinlik [5], Gozde Ozaydin Ince [2,3,6,*] and Ali Zarrabi [1,*]

[1] Biomedical Engineering Department, Faculty of Engineering & Natural Sciences, Istinye University, Istanbul 34396, Turkiye
[2] Faculty of Engineering and Natural Sciences, Materials Science and Nano-Engineering Program, Sabanci University, 34956, Istanbul, Turkiye
[3] Sabanci University Nanotechnology Research and Application Center (SUNUM), Tuzla, Istanbul 34956, Turkiye
[4] TUBITAK Marmara Research Center, Life Sciences Medical Biotechnology, Gebze-Kocaeli, 41470 Turkiye
[5] TUBITAK Marmara Research Center, Life Sciences, Industrial Biotechnology, Gebze-Kocaeli, 41470 Turkiye
[6] Center of Excellence for Functional Surfaces and Interfaces (EFSUN), Sabanci University, Istanbul 34956, Turkiye
* Correspondence: gozdeince@sabanciuniv.edu (G.O.I.), ali.zarrabi@istinye.edu.tr (A.Z.)

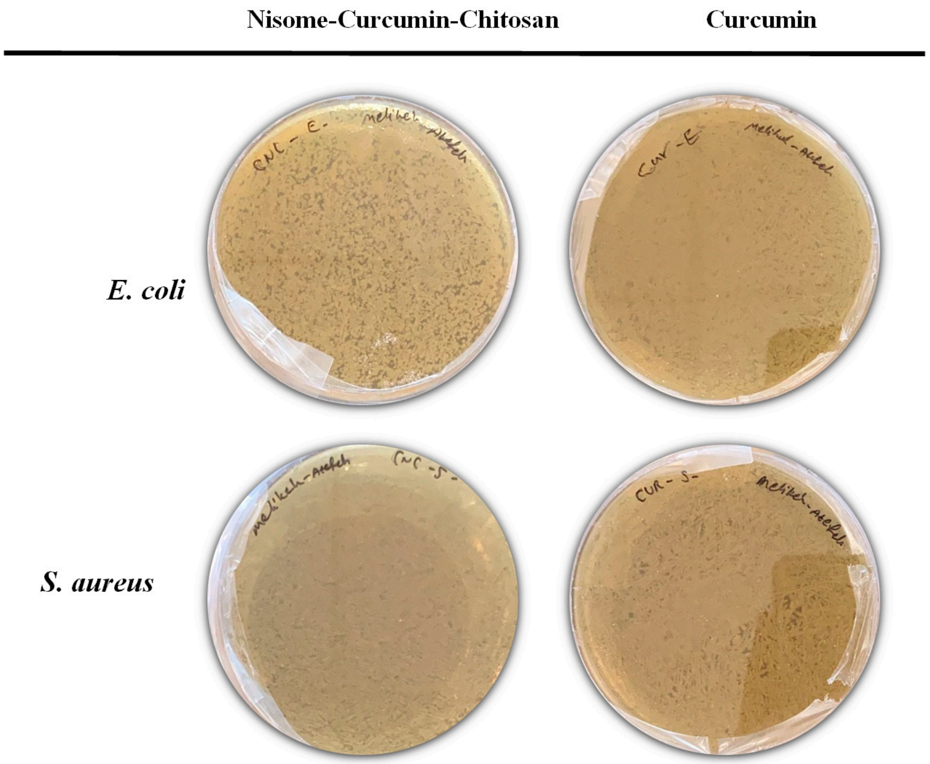

Figure S1. Effects of Curcumin loaded niosome-chitosan nanoparticles and free Curcumin on *E.coli* and *S.aureus*.
